# Meat Composition, Fatty Acid Profile and Sensory Attributes of Meat from Goats Fed Diet Supplemented with Fermented *Saccharina japonica* and *Dendropanax morbifera*

**DOI:** 10.3390/foods9070937

**Published:** 2020-07-15

**Authors:** Jamila Fatima L. Saturno, Muhammad Ammar Dilawar, Hong-Seok Mun, Dae Hun Kim, Dhanushka Rathnayake, Chul-Ju Yang

**Affiliations:** Department of Animal Science and Technology, Animal Nutrition and Feed Science laboratory, Sunchon National University, Suncheon 57922, Korea; jamilasaturno0830@gmail.com (J.F.L.S.); ammar_dilawar@yahoo.com (M.A.D.); mhs88828@nate.com (H.-S.M.); eogns-_-v@naver.com (D.H.K.); dhanus871@gmail.com (D.R.)

**Keywords:** goat meat, fermented feed, meat composition, fatty acids, oxidative stability

## Abstract

A 90-day feeding trial was conducted to evaluate the effects of diets supplemented with three concentrations (control or 0%, 0.5% and 1.0%) of fermented *Saccharina japonica* and *Dendropanax morbifera* (FSJ-DM) on the meat composition, growth performance, oxidative stability and fatty acid profile of Korean native black goat (KNBG) meat. The feed conversion ratio and body weight gain (1st to 2nd month) were improved significantly (*p* < 0.05) in response to feed supplemented with 1.0% FSJ-DM. Moisture content was increased, whereas ether extract and cholesterol contents were decreased in meat obtained from goats supplemented with 1.0% FSJ-DM dietary feeds (*p* < 0.05). In the same diet group, the total saturated fatty acids (ΣSFA) were lower, whereas the sum of polyunsaturated fatty acids (ΣPUFA) was higher, along with higher PUFA/SFA ratio and lower n-6/n-3 ratio (*p* < 0.05). On an average, the dietary supplementation of 1.0% FSJ-DM reduced the thiobarbituric acid reactive substance (TBARS) and pH values of goat meat. Overall, the results of this study suggest that diet supplemented with 1.0% FSJ-DM improves the meat composition, growth performance and fatty acid profile and reduces lipid oxidation of goat meat.

## 1. Introduction

The global human population is predicted to increase up to 9.6 billion by 2050 [1], leading to an increased demand of 70% more livestock production, accounting for 235% more animal feed [2]. Consequently, suitable feed alternatives are being investigated that can enhance the quality of animal feed product, welfare, health, product diversity, environmental protection and economics [3].

Korean native black goat (*Capra hircus coreanae*, KNBG) accounts for more than 80% of the total goat population in South Korea [4]. KNBG meat has a long history and great contribution to local culture, as the ancient Koreans considered it a health food and used it for various medicinal purposes [5]. The consumption of KNBG has significantly increased to 30% during recent years due to increased demand for livestock products [6,7]. KNBG meat is superior in nutritional aspect as compared to beef and pork due to its lower fat content and higher calcium and iron contents [6].

Phytochemicals are defined as organic bioactive compounds produced by plants as part of their normal metabolic activities [8]. The proposed mechanistic actions of herbal feed additives are associated to increased digestibility, modifying of the intestinal microbes and nutrient absorption [9], as well as antibacterial, anti-inflammatory and immunomodulatory properties [10]. *Dendropanax morbifera* (DM) is a perennial tree in Korea, belonging to the ginseng tree family Araliaceae. The leaves of DM have traditionally been used in folk medicine for the cure of different illnesses. The major bioactive components of this plant are flavonoids, polyacetylene compounds, oleifolioside, dendropanoxide and rutin [11]. The methanolic extracts from stems and leaves of DM contain phenolic and flavonoid components and display a strong 2,2-Diphenyl-1-picrylhydrazyl (DPPH) scavenging activity, thereby associating it with high levels of antioxidant activity [12].

There is a growing interest in the utilization of edible seaweeds in the diet of ruminants due to their inherent potential to contribute to the energy and protein requirement of animals [13]. *Saccharina japonica* (SJ) is a macroalgal genus of brown algae, having a high concentration of dietary fibers and protein with a relatively well-balanced profile of amino acids and having an abundant source of bioactive compounds, along with rich contents of vitamins and minerals [14]. Seaweed production is a big industry in Korea, contributing to 6% of the global seaweed aquaculture production [15]. Therefore, its utilization in animal feeds would play an important role in the sustainable development of the livestock sector.

Fermentation of plant materials with probiotics enhances the additional health-promoting features [16,17]. These health-promoting features are beneficial for improving the meat quality and growth performance of animals. Furthermore, plant-based fermented feeds have been well chosen as vectors for inclusion in probiotic cultures. Moreover, it is reported that the probiotic enzyme activity enhances the functionality and sensory value of fermented food products [18]. Various species of the genera *Lactobacillus*, *Bacillus* and *Saccharomyces* remain effective probiotic agents in animal husbandry. These microorganisms have widely adapted to ferment plant materials to manufacture beneficial and practical feed additives for livestock [19,20]. Previous studies have described the effects of fermented feed on Korean native cattle [21]. However, there is insufficient research regarding the supplementation of fermented feed for goats. The principal objective of this feeding trial was to investigate the effects of fermented SJ-DM on the meat quality, growth performance and oxidative stability of KNBG.

## 2. Materials and Methods

### 2.1. Animal Care

This feeding trial was conducted at the Yaksan Black Goat Farm, Jeollanam-do, South Korea. All animals were reared and handled in following with the instructions for the use and care of animals (Ministry for Agriculture, Forestry and Fishery in Korea, 2008).

### 2.2. Fermented Saccharina japonica and Dendropanax morbifera: Preparation, Mixing and Composition

In this experiment, one alga (*Saccharina japonica*) and one herbal plant (*Dendropanax morbifera*) were fermented using *Saccharomyces cerevisiae* KCTC 7904 and *Lactobacillus plantarum* KCTC 3099. SJ and DM leaves were collected from Wando (South Korea). The leaves were cleaned, dried and powdered. The proximate composition of dried SJ and DM was analyzed in triplicate for moisture (method 930.15 using drying oven), crude protein (method 954.01 using Kjeldahl apparatus), crude fat or ether extract content (method 920.39 using Soxhlet apparatus), crude fiber content (method 978.10 using Soxhlet apparatus and furnace) and crude ash content (method 942.05 using furnace), according to the methods described by AOAC [22], and are presented in Table 1. The nitrogen free extract (NFE) was determined by using Equation (1):

% NFE = 100 − (% Crude Protein + % Moisture + % Crude Fiber + % Crude Fat + % Ash)(1)

Fermented feed was prepared by mixing 70% rice bran (as a base) with 20% *Saccharina japonica* and 10% *Dendropanax morbifera* (details about percentage selection in Supplementary Materials Tables S1–S5 followed by inoculating with 30% (*v*/*w*) *L. plantarum* and 30% (*v*/*w*) *S. cerevisiae*. To enable proper fermentation, the mixture was appropriately blended and maintained for 48 h at 37 °C in a fermenter W-1000 (Wonbalhyo Industry Co., Incheon, Korea). Finally, the fermented feed was kept in an airtight plastic bag until combined with the basal diet. The concentration of microorganisms of fermented *Saccharina japonica* and *Dendropanax morbifera* (FSJ-DM) was calculated by mixing 1 g FSJ-DM in 9 mL distilled water. After 1 h, 10-fold serial dilutions using 1 mL of this sample were cultured in agar media, prepared in 0.85% NaCl solution, and incubated at 37 °C for 48 h, after which the no. of colonies were counted. Proximate composition of the FSJ-DM (crude protein, fat, fiber, ash, moisture and NFE) was determined in triplicate (as described earlier) according to the procedure explained by AOAC [22]. The chemical composition of FSJ-DM and the microbial count of fermented feed are presented in Table 2.

### 2.3. Experimental Animals and Diets

Eighteen KNBG (23.50 ± 0.10 kg BW) were reared for a 90-day feeding trial and assigned randomly to one of the three treatments as follows: (i) (control) basal diet + 0% FSJ-DM, (ii) basal diet + 0.5% FSJ-DM and (iii) basal diet + 1% FSJ-DM. Goats were placed in individual pens (1.2 m × 2.5 m) provided with drinker and feeder and subjected to an adaptation period of 7 days before commencement of the experiment. A commercially available concentrate was used as the basal diet, in combination with Timothy grass hay. The concentrate (2% of body weight) and hay (ad libitum) supplemented with FSJ-DM were offered two times a day at 08:00 a.m. and 04:00 p.m. The ingredients and chemical composition of the concentrate and Timothy hay are shown in Table 3.

### 2.4. Growth Performance, Slaughtering and Sampling Procedure

Body weights were measured in the morning before feeding, at the commencement of the study and every subsequent 30 days interval. Feed intake (FI) was determined as a difference between feed offered to goats and feed refusal, and feed conversion ratio (FCR) was calculated as feed per gain.

At the end of the feeding trial, goats were subjected to 12 h starvation, after which the goats were transferred to a commercial abattoir at Suncheon, Jeollanam-do, for slaughtering, according to the slaughterhouse regulations. The left hind legs were separated at the pelvic joint and chilled in the refrigerator for 24 h at 2 °C; then, 250 g of gluteus medius muscle (GM) was sampled from each hind leg (three samples from each animal) for the further analyses. The meat samples were separately vacuum-packed and refrigerated at 4 °C for oxidative rancidity and for other analyses samples were stored at −20 °C, as reported by Ahmed et al. [5].

### 2.5. Sensory Evaluation

Sensory evaluation of the cooked meat samples (approximately 100 g) was performed by 10 trained judges (5 male members and 5 female members of age group 25–35 years) from the Sunchon National University, South Korea. The sensory test facility consisted of individual testing booths with controlled lightening [23]. The samples were provided with a 3-digit code randomly, and serving order was balanced and randomized. After thawing at 4 °C for 24 h, each meat sample (total samples = 18) was cooked on a pre-heated electric grill unit until the internal temperature showed 70 °C reading. The attributes were evaluated using a 7-point scale for color (1: very light/pale, 7: reddish black color), flavor (1: very poor, 7: highly acceptable), tenderness (1: extremely tough, 7: very soft/tender), palatability (1: low palatability, 7: high palatability), juiciness (1: extremely dry, 7: juicy) and overall acceptability (1: highly unacceptable, 7: highly acceptable).

### 2.6. Proximate Composition and Cholesterol Analysis

To calculate the proximate composition of GM, the separable fat and connective tissue were removed manually and thoroughly ground and homogenized with a homogenizer Ultra-Turrax (IKA Werke, GMBH & Co. KG, Staufen, Germany). The moisture (930.15), crude fat (991.36), crude protein (990.03) and crude ash (942.05) contents of the goat meat were determined according to AOAC [22].

Cholesterol content was determined by adding 1 g of each GM sample to reference material (100 µg 5α-cholesterol). After homogenization with 22 mL ethanol and 0.5 N KOH (aqueous), the sample was saponified for 6 h at 23 °C. The total cholesterol was eventually extracted by using hexane and analyzed by gas chromatography (DS-6200: Donam, Seongnam, Gyeonggi-do, South Korea) fitted with a flame ionization detector and a capillary column (Hewlett-Packard, HP-5) of 0.32 mm internal diameter, 30 m length and 0.25 µm polyethene glycol film thickness. Nitrogen gas (N_2_) was used as the carrier. For the first 2 min, the temperature of the oven was maintained at 250 °C and then gradually raised by 15 °C/min until it reached 290 °C (where it was held for 10 min), followed by 10 °C/min increase to 310 °C (final temperature and kept for 10 min). The additional chromatographic parameters were as follows: sample volume injected 2 µL, split ratio 50:1 and injector and detector temperatures at 280 °C. The cholesterol content obtained is presented as mg/100 g meat.

### 2.7. Fatty Acid Composition Analysis

The fatty acid composition of meat was determined (in triplicate) using a direct method for fatty acid methyl ester (FAME) synthesis, as explained by O’Fallon et al. [24], with minor changes. Briefly, in a 15 mL Falcon tube, 0.7 mL of 10 N KOH in water and 6.3 mL methanol was added to 1 g minced meat. The tube was then placed in a water bath for 1.5 h at 55 °C, with strong handshaking for 10 s every 30 min to completely dissolve, hydrolyze and permeate the sample. After placing in a cold tap water bath, 0.57 mL of 24 N H_2_SO_4_ was added and mixed by inversion until K_2_SO_4_ precipitated. After precipitation, the sample was incubated in a water bath for 1.5 h at 55 °C, with vigorous shaking for 10 s every 30 min. After FAME synthesis, the tube was again placed in a cold-water bath. After cooling, 3 mL hexane was added to the sample and centrifuged with Combi-514-R (Hanil, Gimpo, South Korea) at 3000 rpm for 5 min. The top layer (hexane) holding the FAME was dehydrated by passing through Na_2_SO_4_ (anhydrous). The dehydrated and extracted hexane was concentrated up to 1.5 mL, and then put in a GC vial for analysis.

The FAME was analyzed for fatty acid composition with the help of a gas chromatograph (Agilent 7890B, Santa Clara, CA, USA) installed with a Hewlett-Packard HP-88 capillary column (J&W Scientific, Santa Clara, CA, USA) and flame ionization detector, having length 60 m, internal diameter 0.52 and 0.20 µm polyethene glycol-film thickness. An auto-sampler Agilent 7693 (Agilent Technology, Santa Clara, CA, USA) was used to inject samples. The initial temperature of the oven was 125 °C for 1 min, gradually elevated to 145 °C at the rate of 10 °C/min (held for 26 min), then again increased to 220 °C at 2 °C/min and held for only 2 min. Hydrogen gas (H_2_) and purified air were administered as a carrier gas at a flow rate of 400 mL/min and 40 mL/min, respectively, while helium was applied as the makeup gas at a flow rate of 40 mL/min. Both the detector and injector temperature were maintained at 260 °C with the split ratio of 30:1. Fatty acids were recognized by comparing the retention times with standard FAME mixture (Supelco^TM^ 37 Component FAME Mix, 10 mg/mL in CH_2_Cl_2_, Catalogue no. 47885U, Supelco, Bellefonte, PA 16823-0048). Ratios and sum of the fatty acid profile were also calculated, viz., the sum of monounsaturated fatty acids (ΣMUFA), saturated fatty acids (ΣSFA), polyunsaturated fatty acids (ΣPUFA), ratio of unsaturated fatty acids (USFA) to SFA (USFA/SFA) and PUFA/SFA ratio.

### 2.8. Thiobarbituric Acid Reactive Substance (TBARS) and pH Values

The thiobarbituric acid reactive substance (TBARS) values of goat were examined (in triplicate) at 0, 1 and 2 weeks of storage. In short, 4 g meat was added to 10 mL distilled water and 10 mL solution of 20% trichloroacetic acid (TCA) in 2 M phosphoric acid and homogenized for 1.5 min using a homogenizer. The resultant sample mixture was passed through Hyundai Micro No. 60 (Hyundai Co., Ltd. Seoul, South Korea) filter paper. Equal amounts (2 mL) of 2-thiobarbituric acid (98% 4, 6, dihydroxy-2-mercaptopyrimidine, 0.005 M in distilled water) and the filtrate were placed in a shaking water bath for 30 min at 80 °C. Finally, the absorbance was determined at 530 nm using a VIS-Spectrophotometer (Libra S22, Biochrom Ltd. Cambridge, UK), and the TBARS value is expressed as micromoles of malondialdehyde (MDA)/100 g of meat.

The pH was also measured (in triplicate) at 0, 1 and 2 weeks of storage, by mixing 1 g meat with 9 mL distilled water in a homogenizer for 1.5 min, and pH values were determined using a Docu-pH Meter digital pH meter (Sartorius, Goettingen, Germany).

### 2.9. Statistics

The mean values and standard error of the mean (SEM) were calculated. All data were subjected to analysis of variance (ANOVA) using the General Linear Models (GLM) function of the Statistics Analysis System (SAS, 2003, Version 9.1, SAS Institute, Cary, NC, USA). Each pen was served as the experimental unit for growth performance parameters, whereas each animal used as the experimental unit for other parameters. The statistical model used to check the effects of treatment was the following (Equation (2)):

Y_ij_ = µ + α_i_ + e_ij_ (2)

where Y_ij_ is the response variable, µ is the general mean, α_i_ is the effect of dietary treatments, and e_ij_ is the random error. Student’s *t* test was used for comparison between mean values. A *p*-value < 0.05 is considered significant, whereas *p* < 0.10 is defined as a trend.

## 3. Results

### 3.1. Growth Performance

The growth performance of KNBG fed diets supplemented with FSJ-DM is presented in Table 4. Weight gain of KNBG from 1st to 2nd month (*p <* 0.05) were increased in the 1.0% FSJ-DM supplemented diet group and showed a tendency to increase when assessed from 2nd to 3rd month and 0 to 3rd month (*p* < 0.10). The feed intake of KNGB showed no significant difference during the experimental period. However, from 1st to 2nd month, better (*p* < 0.05) FCR was observed in the 1.0% FSJ-DM supplemented group.

### 3.2. Sensory Characteristics and Proximate Composition of Meat

The sensory characteristics (color, flavor, tenderness, juiciness and palatability) are presented in Table 5. The sensory characteristics remained unaffected by dietary treatments (*p* > 0.05).

Moreover, no differences were observed in the crude protein and ash content of meat among treatment groups (Table 6). However, moisture content was increased significantly *(p* < 0.05) as a result of FSJ-DM supplementation. The cholesterol and crude fat content of GM samples were also reduced (*p* < 0.05) in response to 1.0% FSJ-DM supplemented group.

### 3.3. Fatty Acid Composition of Meat

Assessing the SFA content revealed a reduction (*p* < 0.05) in the levels of myristic acid and palmitic acid in meat procured from KNBG fed 1.0% FSJ-DM supplemented diet (Table 7). The total SFA content of GM was also reduced with 1.0% FSJ-DM supplementation. Furthermore, GM samples from the 1.0% FSJ-DM supplemented group showed significantly increased concentrations of linoleic acid, α-linolenic acid, DGLA, arachidonic acid, ΣPUFA and PUFA/SFA ratio and a reduced n-6/n-3 ratio.

### 3.4. Meat pH and Oxidative Stability

**Figure 1:**
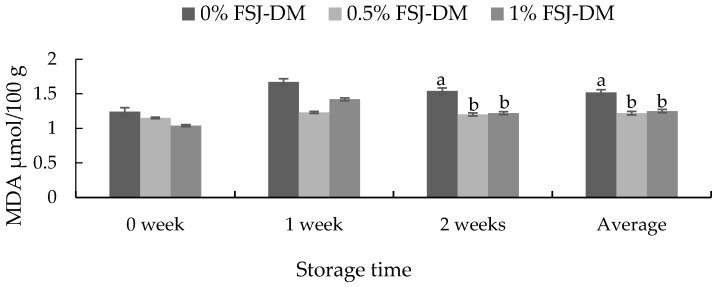
Effects of fermented *S. japonica* and *D. morbifera* (FSJ-DM) on the malondialdehyde (MDA) values in gluteus medius muscle from Korean native black goats. Data are presented as mean ± SE. ^a, b^ Values with different superscripts in the same storage period differ significantly (*p* < 0.05).

The effects of FSJ-DM supplementation on the meat oxidative stability of KNBG are presented in Figure 1. On an average (0–2 weeks) and after 2 weeks of storage, the TBARS value of meat samples from animals supplemented with FSJ-DM was significantly lower (*p* < 0.05) than that from other groups.

**Figure 2:**
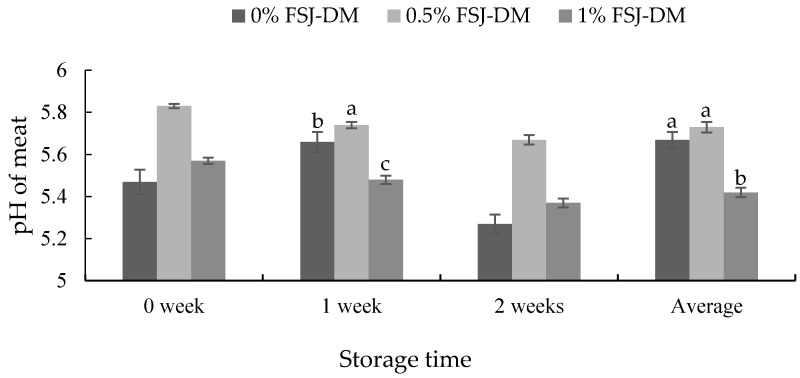
Effects of fermented *S. japonica* and *D. morbifera* (FSJ-DM) on the meat pH from Korean native black goats. Data are presented as mean ± SE. ^a, b, c^ Values with different superscripts in the same storage period differ significantly (*p* < 0.05).

On an average (0–2 weeks) and after 1 week of storage, the pH of KNBG meat was significantly lowered in the 1.0% FSJ-DM supplemented group (*p* < 0.05). However, no differences were observed after 0 and 2 weeks of storage (Figure 2).

## 4. Discussion

Phytochemicals, probiotics, prebiotics and synbiotics not only facilitate the growth performance of animals but also modulate various functions of the body. These feed additives can be suitably utilized by the animal feed industry and hold considerable promise for enhancing livestock production. Furthermore, fermentation of herbs can modify their nutritional composition and bioactivities [25] and intensify the original treatment potency due to enzymes produced by bacteria, molds and yeast [20]. In the present study, 1.0% FSJ-DM showed a tendency to increase the growth performance, oxidative stability and ΣPUFA content, while reducing the cholesterol and ΣSFA.

Improvement in feed efficiency and growth performance of goats fed 1.0% FSJ-DM is thought to be induced by (i) the growth promoting effect of probiotic, including maintenance of beneficial microbial population [26]; (ii) stimulation of digestive secretions due to plant extracts [27] and (iii) improvement in health promoting properties of plants and reduction of anti-nutritional factors due to microbial fermentation [20]. These results are in line with the experiment by Kim et al. [21], who reported that fermented feed improves the nutrient utilization in ruminal fermentation in Korean native cattle. *L. plantarum* and *S. cerevisiae* also produce many extracellular enzymes and possess protease and tannase activities, which may help improve utilization of feed by modifying the gut microflora [28,29]. The better FCR obtained due to consumption of 1.0% FSJ-DM in the current study corroborates the report of Fiesel et al. [30], which states that polyphenols in plants are beneficial in improving the gain/feed ratio in pigs. In addition, the beneficial effects of seaweed supplementation in diets of ruminants on growth performance are also well reported [13,14]. In the present experiment, the non-significant effect on FI in response to FSJ-DM can be described to be due to reduced palatability caused by the existence of complex polyphenols in plants. Jeong et al. [31] also reported that fermented herbs had no significant effect on the feed intake in conjunction with improvement in the gain/feed ratio.

Meat from KNBG has immense market potential due to its inherent rich nutritional aspects as compared to other commonly used red meat sources [6]. In this study, the treatment groups showed no variations in the sensory attributes (color, tenderness, flavor, palatability and juiciness), suggesting that there was no negative impact on the meat consumed. Similarly, Lei et al. [32] reported that fermented herbs exert no effect on the sensory attributes and meat color. Moreover, eating quality parameters by which consumers judge the meat quality are highly subjective, variable and invisible [33].

Dietary supplementation with 1.0% FSJ-DM resulted in reduced crude fat (ether extract) content with simultaneous increase in the moisture content of meat obtained from KNBG. The decreased ether extract could be attributed to the lipolytic activity of flavonoids and polyphenols present in *D. morbifera* [12,34]. Increased moisture content may be because of the inverse relationship between meat moisture and fat content, which, in turn, is associated with meat juiciness [35]. Supplementation with 1.0% FSJ-DM also resulted in reduced meat cholesterol content of KNBG as compared to the control and could be due to the flavonoids present in *D. morbifera.* The flavonoids in plants make insoluble complex with cholesterol and hinder its absorption [20]. Moon [11] first reported the antidiabetic effect of dendropanoxide extracted from the DM leaves, which reduces the serum glucose, triglycerides, and cholesterol levels. It is well known that polysaccharides (fucoidan and alginic acid) in brown seaweed (*Saccharina*) exert a hypoglycemic effect. Alginic acid lowers blood cholesterol levels by producing an indigestible ionic colloid that reduces cholesterol absorption in the gut while increasing fecal cholesterol excretion [36]. Fucoidan, a dietary fiber polysaccharide, binds with bile acids in the small intestine, resulting in fecal excretion and inhibiting reabsorption into the enterohepatic circulation, which in turn causes lowering of bile acids in the liver and, consequently, cholesterol [37]. Furthermore, seaweed from *Saccharina* species is also beneficial in mitigating the stress and anxiety in goats and reducing cholesterol levels [14].

Oxidation in muscle lipids is the cause of production of free radicals, which are implicated in the deterioration of color and flavor in meat. The reaction of malondialdehyde with 2-thiobarbituric acid is extensively used as a reliable marker for determining oxidation in lipids [38]. It is well documented that polyphenols in plants act as antioxidants by scavenging the free radicals and play a vital role in the cellular antioxidant system, preventing oxidative reactions in unsaturated fatty acids [39]. In this experiment, FSJ-DM supplementation significantly decreased the MDA value in meats stored for 2 weeks. The antioxidant potential of *D. morbifera* is due to the existence of higher total phenolic compounds (TPC). Kyung et al. [12] also reported that TPC in *D. morbifera* exhibit a strong correlation with DPPH scavenging ability and reducing power, thereby reducing oxidation reactions in meat. Moreover, supplementation of seaweed potentially increases the superoxide dismutase activity and enhances the antioxidant potential, which helps to combat against oxidative stress and enhances the health status and meat quality of animals [40]. Consistent with our findings, Kannan et al. [41] have also reported that seaweed supplementation helps to boost the antioxidant status of goats. In addition, fermentation of feed with *S. cerevisiae* and *Lactobacillus* spp. exhibit strong radical scavenging ability and reducing power, by increasing the amounts of flavonoids and TPC due to microbial hydrolysis [42,43].

An important indicator of meat quality is the pH, which is also closely related to color and shelf life. Microbiologically, a low pH improves the meat shelf life by inhibiting the putrefactive bacteria. On an average and after 1 week of storage, we observed decreased pH in meats procured from the 1.0% FSJ-DM supplemented group. This can be explained by the various biologically active compounds in *D. morbifera*, including polyphenols, flavonoids, polyacetylene compounds and oleifolioside A, which possess strong antioxidant and antimicrobial properties [44]. These compounds reduce the microbial growth and ultimately reduce the spoilage of meat.

Dietary SFA (palmitic, stearic, myristic acid) increases the cholesterol level and subsequent threat of coronary heart disease. However, PUFA lowers the low density lipoproteins cholesterol and exerts beneficial effects on human health [45,46]. Plant polyphenols have the ability to modify the composition of fatty acids by decreasing the oxidation reactions in unsaturated fatty acids [47]. It is well reported that plant polyphenolic flavonoids protect the UFA against oxidants and could activate antioxidant response element (ARE) mediated gene expressions [48]. In the current study, dietary supplementation with 1.0% FSJ-DM reduced the concentration of saturated fatty acids in GM. Conversely, the concentration of PUFA was higher in the 1.0% FSJ-DM supplemented group. Ahmed et al. [19] and Lei et al. [29] also suggested that fermented herbs modify the composition of fatty acids by reducing the SFA level and increasing the PUFA concentration. The PUFA/SFA and n-6/n-3 ratio is an indicator to evaluate the nutritional value of fat. Fats having high PUFA/SFA and low n-6/n-3 ratio are considered favorable as they decrease the risk of cholesterolaemia [49]. In this study, meat obtained from KNBG supplemented with 1.0% FSJ-DM diet had a lower n-6/n-3 ratio and higher PUFA/SFA ratio, indicating a favorable impact on the quality of meat. It is assumed that the fatty acid composition of GM procured from KNBG fed diet with the addition of 1.0% FSJ-DM may be related to the TPC and antioxidant activity of fermented herbs [31,47]. As an antioxidant, *D. morbifera* and *S. japonica* also prevent the peroxidation of oxidation-liable PUFA in goat meat. Further research is still warranted to identify the exact mechanism of the effects of the dietary supplementation of FSJ-DM on fatty acid composition.

## 5. Conclusions

Results of the present study indicate that meat procured from goats consuming dietary supplementation with 1.0% FSJ-DM increases the weight gain and FCR, with improved nutritional value due to reduced crude fat and cholesterol contents. Furthermore, in addition to low levels of SFA, the improved PUFA content of goat meat was obtained with 1.0% FSJ-DM supplementation. Low pH and TBARS values were clear indications that this treatment is capable of reducing the microbial growth and lipid oxidation in goat meat under refrigerated storage. Considering these results, we conclude that inclusion of fermented feed at levels that are not harmful may be a promising feeding strategy. Further investigation at a commercial farm with a large number of animals is essential to advance methods of enhancing goat productivity through the inclusion of fermented feed.

## Figures and Tables

**Table 1:** Chemical composition (%) of ground plant powder before fermentation.

Parameter	*Saccharina japonica*	*Dendropanax morbifera*	SJ-DM * + Rice Bran
Moisture	6.81 ± 0.10	7.65 ± 0.05	8.64 ± 0.03
Crude protein	8.39 ± 0.06	9.30 ± 0.57	12.58 ± 0.03
Crude fat	3.33 ± 0.12	1.29 ± 0.10	8.04 ± 0.08
Crude fiber	20.96 ± 0.14	6.09 ± 0.16	12.00 ± 0.16
Crude ash	6.92 ± 0.12	23.65 ± 0.09	11.48 ± 0.09
NFE ^1^	53.58 ± 0.31	52.02 ± 0.32	47.26 ± 0.32

* SJ-DM = *Saccharina Japonica* and *Dendropanax morbifera*. The results are reported as mean ± SEM. ^1^ Nitrogen Free Extract.

**Table 2:** Microbial concentration (cfu/g) and chemical composition (%) of fermented *Saccharina Japonica* and *Dendropanax morbifera* (FSJ-DM).

Item	FSJ-DM
Microbial strains	
*Lactobacillus plantarum*	2.03 × 10^8^
*Saccharomyces cerevisiae*	5.93 × 10^6^
Parameter	
Moisture	15.31 ± 0.12
Crude fat	7.22 ± 0.53
Crude protein	12.48 ± 0.07
Crude fiber	12.59 ± 0.55
Crude ash	9.90 ± 0.47
NFE	42.50 ± 0.64

The results are reported as mean ± SEM.

**Table 3:** Composition (%) of the experimental basal diets.

Ingredients (%)	Concentrate	Timothy Hay
Corn grain	16.6	
Wheat Bran	12.0	
Wheat grain	24.9	
Coconut meal	12.0	
Distiller dried grains	8.00	
Palm meal	9.00	
Cottonseed meal	6.50	
Molasses	7.00	
Limestone	2.90	
NaCl	0.60	
Vitamin-mineral premix *	0.50	
Chemical composition (% DM)		
Moisture	12.8	9.2
Crude fat	2.70	2.50
Crude protein	16.0	8.0
Crude ash	8.61	5.0
Crude fiber	7.89	30.0
Acid detergent fiber (ADF)	16.48	35.0
Neutral detergent fiber (NDF)	39.02	62.8
Total digestible nutrients (TDN)	65.6	49.1
Ca	1.20	0.5
Available P	0.48	0.3

* Premix provided the following nutrients per kilogram of diet: vitamin A, 8500 IU; vitamin D3, 800 IU; vitamin E 40 IU; vitamin k, 3.5 mg; Fe, 50 mg; Co, 0.3 mg; Cu, 13 mg; Mn, 40 mg; Zn, 40 mg; I, 0.5 mg; Se, 0.25 mg.

**Table 4:** Growth performance of KNBG fed fermented *S. japonica* and *D. morbifera* (FSJ-DM).

Parameters	0% FSJ-DM	0.5% FSJ-DM	1.0% FSJ-DM	SEM	*p*-Value
	*n* = 6	*n* = 6	*n* = 6		
**0 to 1st month**					
Initial Body Weight (kg)	23.54	23.43	23.48	0.96	1.00
Final Body Weight (kg)	24.57	24.53	24.84	0.67	0.98
Feed Intake (kg)	16.49	16.79	17.25	0.20	0.96
Weight gain (kg)	1.03	1.09	1.36	0.08	0.12
FCR	15.92	15.32	12.62	0.29	0.22
**1st to 2nd month**					
Initial Body Weight (kg)	24.57	24.53	24.84	0.67	0.98
Final Body Weight (kg)	25.51	25.53	26.11	0.42	0.92
Feed Intake	15.10	14.70	14.65	0.44	0.17
Weight gain (kg)	0.94 ^b^	1.00 ^b^	1.27 ^a^	0.06	0.04
FCR	15.57 ^a^	14.62 ^a^	11.48 ^b^	0.36	0.01
**2nd to 3rd month**					
Initial Body Weight (kg)	25.51	25.53	26.11	0.42	0.92
Final Body Weight (kg)	28.61	28.84	29.43	0.56	0.85
Feed Intake	20.60	21.30	20.88	0.46	0.87
Weight gain (kg)	3.10	3.31	3.32	0.24	0.08
FCR	6.60	6.11	6.29	0.35	0.66
**0th to 3rd month**					
Initial Body Weight (kg)	23.54	23.43	23.48	0.96	1.00
Final Body Weight (kg)	28.61	28.84	29.43	0.56	0.85
Feed Intake	50.75	52.10	51.70	0.32	0.25
Weight gain (kg)	5.07	5.40	5.95	0.20	0.06
FCR	10.07 ^a^	9.42 ^a^	8.69 ^b^	0.36	0.04

^a, b^ Values with different superscripts in the same row differ significantly (*p* < 0.05).

**Table 5:** Sensory attributes of meat from KNBG fed fermented *S. japonica* and *D. morbifera* (FSJ-DM).

Parameters	0% FSJ-DM	0.5% FSJ-DM	1.0% FSJ-DM	SEM	*p*-Value
Color	5.21	4.89	5.32	0.86	0.20
Flavor	5.14	4.86	4.79	1.30	0.47
Tenderness	5.64	5.68	5.68	1.69	0.99
Juiciness	5.68	5.61	5.11	1.03	0.08
Palatability	5.68	5.18	5.68	1.63	0.25

Each value represents the mean of 18 replicates (6 goats/treatment and 3 samples/goat).

**Table 6:** Proximate composition and cholesterol content of gluteus medius muscle from KNBG fed fermented *S. japonica* and *D. morbifera* (FSJ-DM).

Parameters	0% FSJ-DM	0.5% FSJ-DM	1.0% FSJ-DM	SEM	*p*-Value
Moisture (%)	77.49 ^b^	78.21 ^a^	78.22 ^a^	0.12	<0.001
Crude protein (%)	22.42	21.15	21.67	0.52	0.10
Crude fat (%)	1.72 ^a^	1.71 ^a^	1.56 ^b^	0.11	0.04
Crude ash (%)	1.16	1.14	1.16	0.01	0.89
Cholesterol (mg/100 g)	92.92 ^a^	85.05 ^a^	69.10 ^b^	1.66	0.002

^a, b^ Values with different superscripts in the same row differ significantly (*p* < 0.05). Each value represents the mean of 18 replicates (6 goats/treatment and 3 samples/goat).

**Table 7:** Fatty acid composition of gluteus medius muscle (g/100 g of total fatty acid) from KNBG fed fermented *S. japonica* and *D. morbifera* (FSJ-DM).

Fatty Acids (g/100 g of Total Fatty Acids)	0% FSJ-DM	0.5% FSJ-DM	1.0% FSJ-DM	SEM	*p*-Value
Capric acid (C10:0)	0.07 ^b^	0.10 ^a^	0.08 ^b^	0.005	0.02
Myristic acid (C14:0)	2.51 ^a,b^	3.07 ^a^	1.92 ^b^	0.66	0.03
Lauric acid (C12:0)	0.19	0.29	0.16	0.01	0.08
Palmitic acid (C16:0)	19.49 ^a^	20.91 ^a^	17.72 ^b^	0.74	0.003
Stearic acid (C18:0)	13.08	13.61	13.35	1.17	0.63
ΣSFA ^1^	35.34 ^a^	37.98 ^a^	33.23 ^b^	0.62	0.01
Palmitoleic acid (C16:1)	2.73 ^a^	2.30 ^a,b^	1.91 ^b^	0.37	0.04
Oleic acid (C18:1)	39.17 ^a^	36.15 ^a,b^	34.01 ^b^	1.27	0.03
Elaidic acid (C18:1 trans)	1.38	1.52	1.69	0.21	0.41
Nervonic acid (C24:1)	0.40 ^b^	0.44 ^b^	0.61 ^a^	0.03	0.04
ΣMUFA ^1^	40.42 ^a,b^	43.68 ^a^	38.22 ^b^	0.36	0.04
Linoleic acid (C18:2 n-6)	6.22 ^b^	6.68 ^b^	9.88 ^a^	0.98	0.01
α-linolenic acid (C18:3 n-3)	0.19 ^b^	0.17 ^b^	0.22 ^a^	0.001	0.01
Eicosadienoic acid (C20:2 n-6)	0.68	0.70	0.92	0.10	0.28
DGLA (C20:3 n-6)	0.27 ^b^	0.30 ^a,b^	0.42 ^a^	0.01	0.05
Arachidonic acid (C18:2 n-6)	3.80 ^b^	3.90 ^b^	6.22 ^a^	0.01	0.02
ΣPUFA ^1^	11.17 ^b^	11.74 ^b^	17.66 ^a^	0.92	0.01
PUFA/SFA	0.33 ^b^	0.31 ^b^	0.54 ^a^	0.02	0.01
n-6/n-3	8.50 ^a^	7.72 ^a,b^	7.06 ^b^	0.70	0.02

^a, b^ Values with different superscripts in the same row differ significantly (*p* < 0.05). ^1^ ΣSFA = saturated fatty acid; ΣMUFA = mono-unsaturated fatty acid; ΣPUFA = polyunsaturated fatty acid; ΣUFA = unsaturated fatty acid. Each value represents the mean of 18 replicates (6 goats/treatment and 3 samples/goat).

## Supplementary Materials

The following are available online at https://www.mdpi.com/2304-8158/9/7/937/s1, Table S1: Treatment compositions for screening of feed additives, Table S2: Proximate composition of milled plant powder mixed with rice bran before fermentation, Table S3: Proximate composition of milled plant powder mixed with rice bran after fermentation, Table S4: Microbiological count of the fermented feed additive treatments, Table S5: Cost of plant-derived feed ingredient per treatment.
