# Supplementary material for: Meat Composition, Fatty Acid Profile and Sensory Attributes of Meat from Goats Fed Diet Supplemented with Fermented Saccharina japonica and Dendropanax morbifera
Source: Foods. 2020 Jul 15;9(7):937. doi: 10.3390/foods9070937 (PMC7404804; doi:10.3390/foods9070937)
Supplement: Supplementary file 1 [file foods-09-00937-s001.pdf]

Table S1. Treatment compositions for screening of feed additives.

Ingredients	Treatments (%)					
	Control	1	2	3	4	5
Saccharina japonica (Dashima)	0	10	10	10	20	30
Dentropanax morbifera(Hwangchil)	0	10	20	30	10	10
Rice bran	100	80	70	60	70	60

Table S2. Proximate composition of milled plant powder mixed with rice bran before fermentation.

Items (%)	Control	T1	T2	T3	T4	T5	SEM	*p*-Value
Moisture	9.75[a]	9.07[b]	9.06[b]	8.64[c]	8.28[d]	8.26[d]	0.0003	<.0001
Crude protein	14.27[a]	13.04[b]	12.84[c]	12.58[d]	12.32[e]	11.91[f]	0.003	<.0001
Ether extract	12.04[a]	9.74[b]	8.79[c]	8.04[d]	7.96[d]	8.18[d]	0.08	<.0001
Crude fiber	11.68[d]	12.84[bc]	11.84[d]	12.00[cd]	13.55[b]	14.56[a]	0.24	<.0001
Crude ash	9.74[d]	10.75[c]	11.28[ab]	11.48[a]	10.89[bc]	9.68[d]	0.07	<.0001
NFE	42.53[c]	44.56[b]	46.19[a]	47.26[a]	47.00[a]	47.41[a]	0.76	<.0001

[a,b,c,d,e,f] Values with different superscripts in the same row differ significantly ($p < 0.05$).

Table S3. Proximate composition of milled plant powder mixed with rice bran after fermentation.

Items (%)	control	T1	T2	T3	T4	T5	SEM	*p*-Value
Moisture	15.76[d]	16.91[b]	17.52[a]	16.79[b]	15.31[e]	16.48[c]	0.01	<.0001
Crude protein	14.44[a]	12.96[b]	12.20[d]	11.56[e]	12.48[c]	11.56[e]	0.004	<.0001
Ether extract	10.28[a]	7.52[b]	6.60[c]	6.07[c]	7.22[b]	6.43[c]	0.11	<.0001
Crude fiber	10.28[b]	10.45[b]	11.09[b]	10.62[b]	12.59[a]	13.30[a]	0.22	<.0001
Crude ash	9.96[c]	9.69[c]	10.78[b]	12.24[a]	9.90[c]	8.98[d]	0.05	<.0001
NFE	39.27[c]	42.47[ab]	41.82[b]	42.72[ab]	42.50[ab]	43.25[a]	0.48	0.0002

[a,b,c,d,e,f,] Values with different superscripts in the same row differ significantly ($p < 0.05$).

Table S4. Microbiological count of the fermented feed additive treatments.

Treatments	Probiotic Bacteria (cfu/g)	
	Lactobacillus plantarum	*Saccharomyces cerevisiae*
Control	2.26×10^{8} [a]	3.24×10^{7} [a]
T1	3.50×10^{7} [b]	1.22×10^{6} [b]
T2	7.87×10^{7} [b]	7.37×10^{6} [b]
T3	3.60×10^{7} [b]	2.51×10^{6} [b]
T4	2.03×10^{8} [a]	5.93×10^{6} [b]
T5	2.13×10^{8} [a]	8.40×10^{5} [b]
p-Value	0.001	<.0001

Table S5. Cost of plant-derived feed ingredient per treatment.

Treatments	Price (KRW)			
	Rice Bran	*S. japonica*	*D. morbifera*	Total Cost for 1 kg
Control	250	0	0	250
T1	200	700	3000	3900
T2	175	700	6000	6875
T3	150	700	9000	9850
T4	175	1400	3000	4575
T5	150	2100	3000	5250

1 KRW = 0.00082 USD; 1 USD = 1235 KRW.
